# Interannual variation in foraging decisions in chick-rearing black-legged kittiwakes

**DOI:** 10.1093/beheco/araf018

**Published:** 2025-03-12

**Authors:** Philip Bertrand, Joël Bêty, Nigel Gilles Yoccoz, Mikko Vihtakari, Kyle Elliott, Stephanie M Harris, Samantha C Patrick, Hallvard Strøm, Harald Steen, Sébastien Descamps

**Affiliations:** Départment de Biologie, chimie et géographie and Centre d’études nordiques, Université du Québec à Rimouski, 300 Allée des Ursulines, Rimouski G5L 3A1, Canada; Norwegian Polar Institute, Fram Centre, 6606 Stakkevollan,Tromsø 9296, Norway; Départment de Biologie, chimie et géographie and Centre d’études nordiques, Université du Québec à Rimouski, 300 Allée des Ursulines, Rimouski G5L 3A1, Canada; Department of Arctic and Marine Biology, UiT The Arctic University of Norway, 6050 Stakkevollan, Tromsø 9037, Norway; Institute of Marine Research, Fram Centre, 6606 Stakkevollan,Tromsø 9296, Norway; Department of Natural Resource Sciences, McGill University, 21111 Lakeshore Road, Ste. Anne de Bellevue H9X 3V9,Canada; School of Environmental Sciences, University of Liverpool, Brownlow Hill, Liverpool L69 7ZX, United Kingdom; Cornell Lab of Ornithology, Cornell University, 159 Sapsucker Woods Rd., Ithaca 14850, United States; School of Environmental Sciences, University of Liverpool, Brownlow Hill, Liverpool L69 7ZX, United Kingdom; Norwegian Polar Institute, Fram Centre, 6606 Stakkevollan,Tromsø 9296, Norway; Norwegian Polar Institute, Fram Centre, 6606 Stakkevollan,Tromsø 9296, Norway; Norwegian Polar Institute, Fram Centre, 6606 Stakkevollan,Tromsø 9296, Norway

**Keywords:** Arctic, bimodal foraging strategy, biologging, central place foraging, foraging decisions, *Rissa tridactyla*

## Abstract

Long-lived species must balance allocation between reproduction and self-maintenance, and such a trade-off is expected to affect their foraging behavior. A bimodal foraging strategy, where individuals alternate between long trips for self-maintenance and short trips for offspring provisioning, may reflect this compromise. Using tracking data collected over three breeding seasons, we investigated the occurrence of a bimodal foraging strategy and inter-annual variation in foraging decisions among black-legged kittiwakes (*Rissa tridactyla*) breeding in Kongsfjorden, Svalbard. Kongsfjorden, a glacial fjord with six tidewater glacier fronts, provides close foraging opportunities to breeding sites. The continental shelf break outside the fjord offers another foraging area but involves higher commuting costs. We tested the hypothesis that breeding adults perform foraging trips outside the fjord for self-maintenance. We predicted that (1) adults were more likely to undertake foraging trips outside the fjord when their body condition was low and that (2) individuals foraging outside the fjord were likelier to improve their body condition than those foraging within. Our results indicate that kittiwakes in Kongsfjorden may adopt a bimodal foraging strategy during chick-rearing, but not every year. Contrary to our first prediction, we found no evidence that adult body condition affected the probability of foraging at distant sites. However, adults were more likely to maintain or improve body condition during outside-fjord foraging trips, supporting the hypothesis that long-distance trips can be used for self-maintenance. Overall, our results suggest that bimodal foraging is not a fixed characteristic of kittiwake foraging behavior and may be influenced by environmental conditions.

## Teaser

Black-legged kittiwakes are expected to balance self-care and provisioning for their offspring through flexible foraging behavior. By studying their movements over three breeding seasons in Kongsfjorden, Svalbard, we hypothesized that birds in poorer body conditions would undertake longer trips to high-quality habitats for self-care. Surprisingly, body condition did not influence trip length. However, longer trips were associated with maintaining or improving body condition. This suggests that their foraging strategy is dynamic and influenced by the environmental conditions in the fjord.

## Introduction

Central place foraging is common across a wide range of terrestrial and marine- taxa (eg Kacelnik 1984; Kacelnik et al. 1986; Fryxell and Doucet 1991). By definition, central place foragers must periodically return to a central location after each foraging bout (Orians and Pearson 1979). Theoretical models of central place foraging assume that resources are patchily distributed in space and that foraging decisions are based on trade-offs between the quality and the distance of foraging patches. These models suggest that foraging costs increase with increasing distance from the central base, and benefits increase with patch quality (Orians and Pearson 1979; Schoener 1979). Central place foragers are thus predicted to forage in the nearest suitable patches.

During the breeding season, central place foragers allocate foraging efforts to both self-maintenance and chick provisioning. Travelling long distances may allow animals to exploit high-quality patches and improve their body condition, yet longer trips may also jeopardize offspring growth or survival via reduced food delivery rate (Ydenberg 1994; Ydenberg and Davies 2010). When foraging takes place near the breeding site, offspring may benefit from a higher feeding rate; however, nearby feeding patches may not necessarily be optimal for individuals’ self-maintenance. Consequently, the same food patches may not be optimal for both adult self-maintenance and offspring provisioning. Hence, the optimal foraging decisions of central place foragers may reflect trade-offs between individual self-maintenance and allocation to reproduction, and these decisions may vary during the breeding season because nutritional and/or energetic requirements vary with offspring age (Murphy 1996; Markman et al. 2004).

The foraging behavior of breeding animals is expected to be dependent on their body condition (eg Varpe et al. 2004) but such relationships can vary according to the life history of the species. In long-lived species, individuals in poor condition are expected to prioritize their own survival over that of their offspring (Clutton-Brock 1988; Newton 1989). If optimal foraging patches which allow central place foragers to improve their body condition are further from their nesting site, individuals in poor condition are expected to perform longer foraging trips to reach these patches than those in good condition (Ydenberg and Davies 2010; Welcker et al. 2012). Such decisions may lead to the adoption of a bimodal foraging strategy, whereby breeding individuals alternate between short foraging bouts to maximize offspring provisioning rates and long foraging bouts to improve their own body condition (Chaurand and Weimerskirch 1994; Weimerskirch et al. 1994; Baduini and Hyrenbach 2003; Ydenberg and Davies 2010). Such a bimodal foraging strategy has been well documented in seabirds, especially in Procellariiformes (eg Chaurand and Weimerskirch 1994; Baduini and Hyrenbach 2003) and Charadriiformes (eg Steen et al. 2007; Welcker et al. 2009, 2012; Elliott and Gaston 2015). Although short-term benefits of long foraging trips have been reported in colonial seabirds (eg increase adult body condition), unraveling how these advantages are impacted by annual changes in environmental conditions poses a persistent challenge (see Granadeiro et al. 1998; Ballard et al. 2010; Jakubas et al. 2020; Fayet et al. 2021; Gee et al. 2024). Interannual variations in prey abundance and distribution are likely to affect both the benefits and the necessity of undertaking long foraging trips (Granadeiro et al. 1998; Ballard et al. 2010). If patches that allow central place foragers to cover both self-maintenance and chick provisioning are located near the nesting site, individuals could limit themselves to shorter foraging trips.

The black-legged kittiwake (*Rissa tridactyla,* hereafter kittiwake) is a long-lived seabird known to use a bimodal foraging strategy during the breeding period (Kotzerka et al. 2010; Paredes et al. 2012; Christensen-Dalsgaard et al. 2018), and whose foraging behavior can be affected by inter-annual changes in oceanographic conditions (Goutte et al. 2014; Paredes et al. 2014; Bertrand et al. 2021a). Kittiwakes breeding in Kongsfjorden, a glacial fjord located on the west coast of Spitsbergen, Svalbard (78.91°N, 11.93°E), can undertake long trips to forage in the open sea but can also forage extensively at tidewater glacier fronts near their colonies (Urbański et al. 2017; Nishizawa et al. 2020; Bertrand et al. 2021b; Stempniewicz et al. 2021). Glacier fronts are spatially restricted and relatively predictable foraging patches (Lydersen et al. 2014; Urbański et al. 2017), however, their profitability appears to vary among years (Stempniewicz et al. 2017; Dragańska-Deja et al. 2020; Bertrand et al. 2021a). Kongsfjorden has no sill and is directly connected to the continental slope, making it influenced by both the Arctic waters from the coastal current and the Atlantic waters from the West-Spitsbergen current (Svendsen et al. 2002; Hop et al. 2019). The intrusions of these water masses vary annually (Cottier et al. 2005; Tverberg et al. 2019) and influence the level of nutrients and the abundance and distribution of kittiwake prey species in the fjord (Vihtakari et al. 2018; Hegseth et al. 2019; Hop et al. 2019).

In this study, we used fine-scale GPS tracking data of chick-rearing kittiwakes collected over three breeding seasons in Kongsfjorden to investigate the interannual variation in their foraging decisions, in which adults can alternate between short (ie inside-fjord) and long (ie outside-fjord) foraging trips (Harris et al. 2020; Bertrand et al. 2021b). We aimed to test the hypothesis that breeding adults perform outside-fjord foraging trips for self-maintenance during the chick-rearing period. We then predicted that 1) outside-fjord foraging trips are more likely to occur when adult body condition is low and that 2) adults performing outside-fjord foraging trips are more likely to improve their body condition than those foraging mainly within the fjord. As environmental conditions encountered during the breeding season can affect bird conditions and foraging patterns (eg Weimerskirch et al. 2001; Christensen-Dalsgaard et al. 2018), we also quantified the annual abundance of food resources using estimates of zooplankton biomass in Kongsfjorden (Hop et al. 2019).

## Methods

### Study system and species

**Fig. 1.**
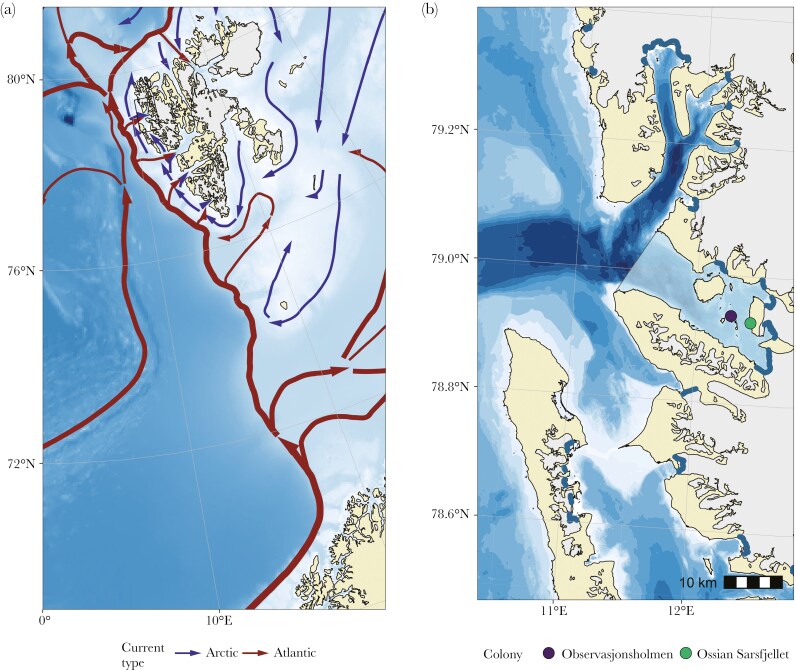
Overview of the study area. The Norwegian Sea and Svalbard (a). The Atlantic currents, located between 200 and 600 m depth, are shown using red arrows highlighting two main entryways: along the continental slope and in the middle of the Norwegian Sea along the Knipovitch ridge. The Arctic surface currents, shown using blue arrows, originate from the Arctic Ocean. The black rectangle indicates Kongsfjorden. Spatial extent of the study area highlighting the boundary of the fjord (light blue shaded area; Kongsfjorden, Svalbard) (b). Blue-shaded areas represent glacier fronts and circles breeding colonies of kittiwakes targeted by this study (purple = Observasjonsholmen; green = Ossian Sarsfjellet).

This study was conducted in the summers 2016, 2017 and 2018 at two kittiwake breeding colonies within Kongsfjorden, namely Observasjonsholmen (hereafter OBS; 78.93°N, 12.28°E) and Ossian Sarsfjellet (hereafter OSS; 78.92°N, 12.44°E; Fig. 1). There are six tidewater glacier fronts in the fjord, which are all used to a varying extent by breeding kittiwakes during the incubation and chick-rearing periods (Bertrand et al. 2021a).

The kittiwake is a surface-feeding seabird species, feeding on small fishes and crustaceans (Coulson 2011; Vihtakari et al. 2018). Kittiwakes breed monogamously once a year and lay one to three eggs per clutch (generally one or two on Svalbard). Hatching occurs in early/mid-July (Burr et al. 2016). Both parents exhibit parental care and feed offspring until fledging, which occurs around 40 d after hatching (Coulson 2011).

### Field data collection

We captured adult kittiwakes on their nest using a noose pole. Nest content was assessed and only birds having at least one chick in their nest were equipped with a GPS logger. Upon capture, we measured head-bill length to the nearest millimeter using a caliper and weighed each individual to the nearest 5 g using a 600 g Pesola scale. Birds were weighed a second time at logger retrieval (i.e recapture) 2 to 6 d after the initial capture. Three types of loggers were deployed in this study: i-gotU GT-120, Mobile Action; CatLog Gen1, and CatLog Gen2. All devices were sealed in waterproof tubing and then fitted on birds’ back feathers using TESA tape. Loggers weighed on average 11.8 g (range = 7.2 to 18.4; representing 2% to 5% of birds’ mass). Deployments occurred between 28 June and 3 August. We found no effect of relative logger weight on the probability of foraging outside the fjord or on the relative body mass changes in birds breeding in Kongsfjorden (see details in Supplementary material 1). In total, 71 chick-rearing adult kittiwakes were tracked (Table 1). Among these, 6 individuals (*ca.* 9%) were equipped more than once (either during the same year or in different years).

**Table 1.** Annual number of foraging trips and adult black-legged kittiwakes (in parentheses) tracked during the chick-rearing period in two colonies located in Kongsfjorden, Svalbard. A total of 71 individuals were captured and six of them were tracked more than once (see methods).

Colony	2016		2017		2018	
	Female	Male	Female	Male	Female	Male
Ossian Sarsfjellet	23 (7)	38 (9)	18 (3)	26 (5)	23 (7)	23 (5)
Observasjonsholmen	54 (9)	54 (11)	50 (7)	36 (6)	14 (4)	15 (5)
Total	77 (16)	92 (20)	68 (10)	62 (11)	37 (11)	38 (10)

The sex of individuals was determined based on a molecular approach using DNA from either feather material or blood samples (*n* = 67, *ca.* 94%; see details in Harris et al. 2020). Otherwise, sex classification was performed using head-bill length morphometric cutoff (x) of 90.5 mm (female ≤ x < male; Coulson 2009) when DNA material was unavailable (*n* = 4, *ca.* 6%). In total, 35 females and 36 males were tracked.

Tracking periods varied among individuals (mean = 69.9 h, range = 42.9 to 142.6 h) and tracking intervals ranged from 2 to 10 min but were subsampled to obtain a standardized resolution of 10 min between successive locations. Based on previous work, we considered an individual to be foraging if it flew farther than 200 m from its colony center and for at least 50 consecutive minutes (Bertrand et al. 2021a). Since kittiwakes spend time on land to fetch nesting materials or to bathe in freshwater (Coulson and Macdonald 1962), we excluded trips that had more than 50% of their locations overlapping land to avoid analyzing non-foraging trips. About 8% of the trips were incomplete (ie last locations being outside the colony) due to battery failure. Among those incomplete trips, we only retained trips where birds had completed the majority of their foraging trip (ie when they returned towards the colony and had traveled at least 75% of the maximum distance reached on their trip; eg Paredes et al. 2012; Harris et al. 2020). A total of 374 trips were delineated from the raw tracks (Table 1), ranging from 1 to 16 per individual (mean ±SD; 5.3 ± 3.3).

### Zooplankton biomass

Prey abundance and distribution are known to vary annually in Kongsfjorden (Vihtakari et al. 2018; Hop et al. 2019). To investigate the link between the foraging patterns of birds and resource abundance, we used seasonal zooplankton biomass in Kongsfjorden estimated from a long-term monitoring program (Hop et al. 2019). Five stations, distributed along a transect from the inner to outer fjord have been systematically sampled during the study period (2016 to 2018) between 13 and 31 July. Zooplankton were sampled using a multiple plankton sampler (MultiNet type Midi, Hydro-Bios), having five closing nets with an opening of 0.25 m^2^ and a mesh size of 200 µm. The closing nets of the multiple plankton sampler were operated at the following depths: seafloor, 200, 100, 50, 20 m and at the surface. The biomass index was calculated as the depth-strata averaged abundance (ind m^-3^) of each species consumed by kittiwakes in Kongsfjorden and then converted to a dry biomass estimate (mg dry mass m^-3^). Estimates from each station were averaged to obtain a yearly seasonal biomass index at the Kongsfjorden level. Details of the procedure are available in Hop et al. (2019) and Bertrand et al. (2021a).

### Data analyses

#### Annual variation in foraging behavior.

We first assessed whether the presence of a bimodal foraging strategy could be detected each year in the kittiwake population breeding in Kongsfjorden. We used the Hartigans’ dip test from the diptest package (version 0.76-0; Ameijeiras-Alonso et al. 2021) to evaluate whether distributions of maximum foraging ranges (ie maximum distance traveled from the colony, which is highly correlated to trip duration, Pearson’s *r* = 0.81) followed a bimodal pattern. This test evaluates the null hypothesis that the distribution is unimodal, against the alternative hypothesis that the distribution is multimodal. To avoid pseudo-replication in these analyses, we, here, used the maximum range reached by an individual during its entire tracking period, resulting in one value per individual (and not one value per trip, which was used in the remaining analyses – see below). Only the first tracking period was considered when individuals were tracked multiple times in a given season. We then estimated the locations of modes in each distribution (ie year) using the critical bandwidth proposed by Hall and York (2001). Limits for the calculation of the critical bandwidth were set between 0 and 200 km to avoid the identification of artificial modes in the right tail of distributions (Ameijeiras-Alonso et al. 2021). To assess the robustness of these results, we conducted a second analysis using a resampling approach. This method accounted for the probability of including all trips made by each individual. Across 999 iterations, we randomly selected one single trip per individual for each year and extracted its maximum foraging range. We then calculated the corresponding *p*-values using the Hartigans’ dip test, creating a probability distribution of *p*-values for each year. Bimodality was then validated based on the visual inspection of the characteristics of these distributions (Murdoch et al. 2008).

We used a mixed modeling approach to investigate whether maximum ranges of both inside- and outside-fjord foraging trips were consistent over the years. Delimitation of the inside-fjord area was based on the extent of the middle and inner zones of Kongsfjorden (Hop et al. 2002), corresponding to a surface area of ca. 230 km^2^ with outside borders delineated by a transect from Kapp Guissez to Kvadehuken (Fig. 1). Maximum range distributions of both groups (ie inside and outside-fjord foraging trips) were highly right-skewed and were thus log-transformed to improve linearity with predictors. Estimates of predicted values were then back-transformed to ease interpretation, corresponding to the median of the response distribution on the original scale. In addition to the year, the colony was added as a predictor to account for the average difference in habitat use among colonies (Bertrand et al. 2021b). We fitted individual identity as a random intercept to account for the non-independence of repeated individual measurements. The model was defined as follows:


$$\begin{array}{lll} log & \left(maximum\;range\right)\;∼\;\alpha +\beta_{1}\left(year_{2017}\right)\; & +\beta_{2}\left(year_{2018}\right)+\beta_{3}\left(colony_{Ossian\;Sarsfjellet}\right)+\left(id\right), \end{array}$$

with α corresponding to the intercept (corresponding to the year 2016 and Observasjonsholmen colony as reference levels), $\beta_{i}$ the different predictors (defined as contrasts) and (id) the random individual effect. Modelling was performed under a Bayesian framework using the rstanarm package (version 2.21.1; Goodrich et al. 2020). We used default weakly informative prior distributions for each parameter to regularize computation and prevent model overfitting (Muth et al. 2018). We performed five Markov chains of 60,000 iterations, including 30,000 iteration-warmups that were subsequently discarded before the estimation of the parameters’ posterior distributions. All effective sample sizes ($N_{eff}$ > 1000) and potential scale reduction factors ($\hat{R}$ < 1.1) indicated model convergence (Muth et al. 2018). Along with estimates, we reported the 95% posterior uncertainty interval (ie 95% PI) of the posterior probability distribution for each model parameter and considered we had evidence for an effect if this interval was not overlapping zero. Posterior predictive checking (ie comparison between predictive distribution and observed data) was performed visually to assess the goodness-of-fit of models.

We used a generalized linear mixed model with a binomial distribution and a logit link function to test for annual variation in the probability of performing outside-fjord foraging trips. Foraging trips were coded as binary, where 0 was assigned to trips where birds remained inside fjord boundaries to forage (ie inside-fjord; light blue area depicted in Fig. 1) and 1 to trips where birds flew out of the fjord (ie outside-fjord). We added year and colony as predictors, and the individual identity was fitted as a random intercept, giving the following form:


$$\begin{array}{lll} log\left[\frac{P}{1-P}\right]∼\;\alpha & +\beta_{1}\left(year_{2017}\right)+\beta_{2}\left(year_{2018}\right)\; & +\beta_{3}\left(colony_{Ossian\;Sarsfjellet}\right)+\left(id\right), \end{array}$$

with P corresponding to the probability of foraging outside the fjord, α the intercept, $\beta_{i}$ the different predictors and (id) the random effect.

#### Linking initial body condition and mass change to foraging decision.

Generalized linear mixed models with a binomial distribution and a logit link function were used to test the prediction that outside-fjord foraging trips were more likely to occur when the adult body condition was low. We coded the response variable based on the first trip performed by the bird after capture and assigned 0 if the bird performed an inside-fjord foraging trip and 1 for an outside-fjord trip. We defined body condition as the size-corrected mass (Jakob et al. 1996; Jacobs et al. 2012) calculated as the residuals of the linear relationship between individual mass and total head-bill length. This metric is expected to represent the individual nutritional status (ie a proxy of the level of nutrient stores). The total head-bill length is a proxy for the structural body size that allows differentiating sexes (estimate of the head-bill length effect on body mass = 5.96, 95% CI = 3.22 to 8.71; *R*^*2*^ = 0.20; *df* = 76; Coulson 2009; Jacobs et al. 2012). Colony was also added as a predictor to account for potential inter-colony differences in foraging behavior (Bertrand et al. 2021b) and the individual identity as a random intercept. Including sex in the analysis did not affect parameter estimates (data not shown). The model was computed as follows:


$$\begin{array}{lll} log\left[\frac{P}{1-P}\right]∼\;\alpha & +\beta_{1}\left(BC\right)+\beta_{2}\left(I\left(year_{2016}\right)\right)+\beta_{3}\left(I\left(year_{2017}\right)\right)\; & +\beta_{4}\left(I\left(year_{2018}\right)\right)+\beta_{5}\left(colony_{Ossian\;Sarsfjellet}\right)+\left(id\right), \end{array}$$

with P corresponding to the probability of foraging outside the fjord, α the intercept, $\beta_{i}$ the different predictors (BC corresponding to body condition, $I\left(year_{20XX}\right)$ an indicator variable for year 20XX (XX = 16, 17 and 18)), and (id) the individual random effect. Then, we used linear mixed models to test the prediction that individuals performing outside-fjord foraging trips were more likely to improve their body condition compared to those foraging mainly within the fjord. The response variable was the relative body mass change (BMC) between the initial capture and recapture;


$$\begin{array}{l} BMC=\frac{\left(M_{r}-M_{c}\right)}{M_{c}} \end{array}$$

where M corresponds to the mass at capture ($M_{c}$) or recapture ($M_{r}$). As a predictor of BMC, we initially aimed at using the foraging site location (ie inside- vs outside-fjord) of birds on their last trip before being recaptured. However, many tracking periods were incomplete due to battery failure which constituted a limited sample size. As an alternative, we used the proportion of outside-fjord foraging trips among all trips performed during the tracking period (ie between capture and recapture). In parallel, we tested the robustness of the results by using a binary predictor (ie instead of the proportion), coded as 0 if the birds stayed inside the fjord during the entire tracking period, and 1 otherwise. Similar results were obtained using this alternative predictor (results not shown). We, thus, only provided results based on the proportion of outside-fjord foraging trips as a predictor of the BMC. Like previous models, we also added the colony as a predictor to the model and the individual identity as a random intercept, giving the following form:


$$\begin{array}{llll} BMC\;∼\;\alpha & +\beta_{1}\left(\;proportion\right)+\beta_{2}\left(I\left(year_{2016}\right)\right)\; & +\beta_{3}\left(I\left(year_{2017}\right)\right)+\beta_{4}\left(I\left(year_{2018}\right)\right)\; & +\beta_{5}\left(colony_{Ossian\;Sarsfjellet}\right)+\left(id\right), \end{array}$$

with α corresponding to the intercept, $\beta_{i}$ the different predictors (proportion indicating the proportion of outside-fjord foraging trips), $I\left(year_{20XX}\right)$ an indicator variable for year 20XX (XX = 16, 17 and 18), and (id) the individual random effect.

## Results

**Fig. 2.**
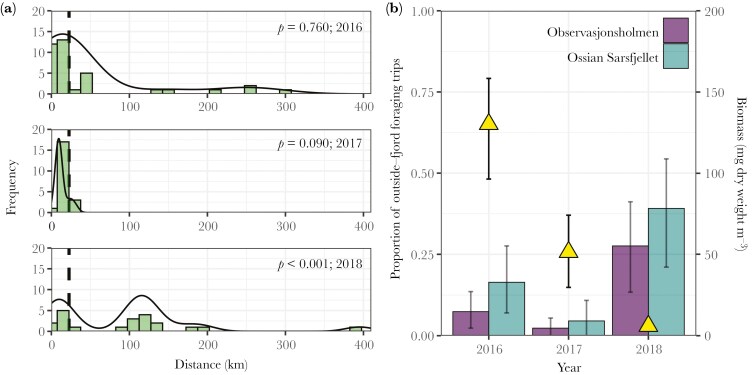
Interannual variation in the frequency distribution of the maximum foraging ranges (km) in chick-rearing kittiwakes breeding in Kongsfjorden (a), and the proportion of foraging trips performed outside of the fjord for each colony, as well as the seasonal zooplankton biomass density (yellow triangle) (b). In panel a, the solid black line represents density distributions of observations and the dashed line corresponds to the distance threshold separating the fjord from the open sea. The Hartigans’ dip test p-values are also indicated for each year. Average annual zooplankton biomass and 95% confidence intervals were calculated by bootstrapping station estimate along 999 iterations using the Hmisc package (version 4.7-2; Harrell et al. 2020). The average annual and colony-specific proportion of outside-fjord foraging trips were calculated using individual proportions but weighted values using the number of trips recorded per individual. The displayed 2.5 and 97.5% percentiles for each average were computed by bootstrapping individual proportions and associated weights along 999 iterations via the boot package (version 1.3-25; Canty and Ripley 2024).

**Fig. 3.**
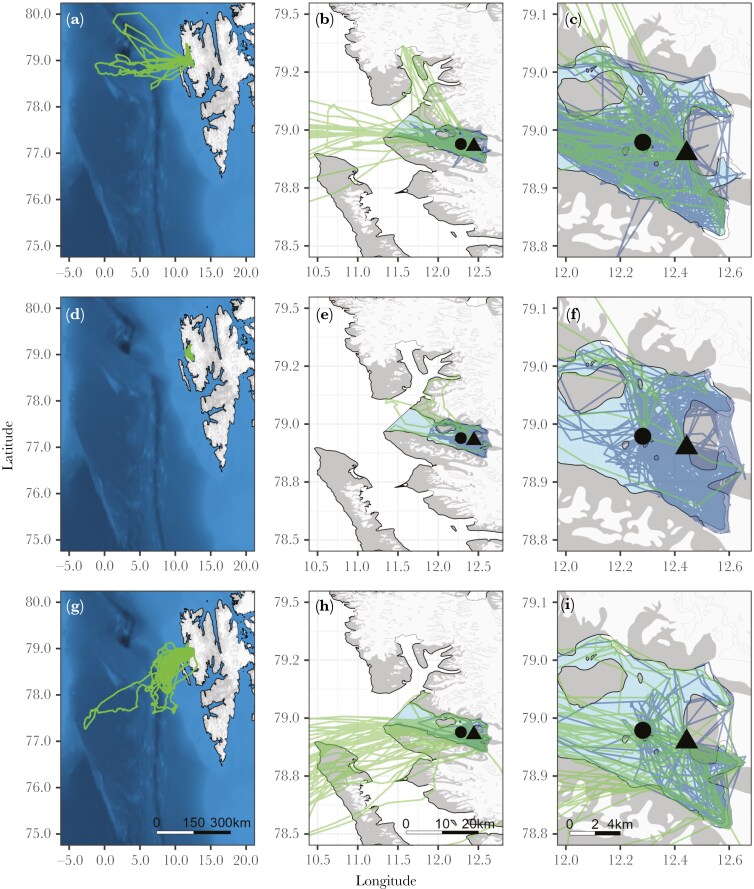
Foraging trips of the 71 chick-rearing adult kittiwakes breeding in Kongsfjorden and tracked with GPS in 2016 (a, b, c), 2017 (d, e, f), and 2018 (g, h, i). Light-green tracks represent outside-fjord foraging trips and dark-blue tracks inside-fjord foraging trips. The location of the two colonies (Observasjonsholmen = black circle; and Ossian Sarsfjellet = black triangle) is also provided. Three spatial scales are shown: left = western Svalbard; center = Kongsfjorden and Krossfjorden area and right = Kongsfjorden.

Chick-rearing kittiwakes breeding in Kongsfjorden foraged from 3.8 to 395.0 km from their colony (Figs. 2a and 3). The distribution of maximum foraging ranges followed a bimodal pattern in 2018 (*D* = 0.15; *p* = < 0.001; mode_1_ = 10.0 km; mode_2_ = 115.8 km) while being unimodal in 2016 (*D* = 0.05; *p* = 0.760; mode = 14.4 km) and 2017 (*D* = 0.10; *p* = 0.090; mode = 9.5 km). Further analysis by resampling confirmed these annual differences in the distribution of maximum foraging ranges (see Supplementary Information 2). Nonetheless, some outside-fjord foraging trips were observed in all years and the maximum foraging range reached during these trips was significantly longer on average than inside the fjord (mean difference in 2016 = 105.9 km, 95% PI = 91.5 to 120.6; 2017 = 19.2 km, 95% PI = 17.1 to 21.2; 2018 = 120.9 km, 95% PI = 98.4 to 143.7). Maximum foraging ranges of inside-fjord trips were similar in all years (median in 2016 = 5.2 km, 95% PI = 4.3 to 6.1; 2017 = 6.7 km, 95% PI = 5.6 to 8.1; 2018 = 5.6 km, 95% PI = 4.4 to 7.2), while ranges of outside-fjord foraging trips were significantly smaller in 2017 (median = 23.3 km, 95% PI = 11.6 to 45.9) compared to 2016 and 2018 (median in 2016 = 75.1 km, 95% PI = 48.3 to 115.9; 2018 = 90.2 km, 95% PI = 59.4 to 138.0). Importantly, outside-fjord foraging trips were not restricted to the use of the open sea. In 2016 and 2017 (1% to 4% of trips per year), birds reached glacier fronts located in an adjacent fjord (Krossfjorden), up to *ca.* 50 km from Kongsfjorden (Fig. 1).

The proportion of outside-fjord foraging trips varied significantly among years (Fig. 2b), being higher in 2018 (31%, 95% PI = 14 to 64) compared to 2017 (2%, 95% PI = 0 to 5) and 2016 (7%, 95% PI = 3 to 14). The proportions of outside-fjord foraging trips observed in 2016 and 2017 were not significantly different (log-odds = 0.8, 95% CI = −1.9 to 3.5). Interestingly, 2018 was characterized by the lowest level of zooplankton biomass in Kongsfjorden (Fig 2b). Individual body conditions at capture also differed among years, being the highest in 2017 (mean = 22.2 g mm^−1^, 95% PI = 8.6 to 35.7), and similar in 2016 and 2018 (mean 2016 = −9.0 g mm^−1^, 95% PI = −20.5 to 2.6; mean 2018 = −13.7 g mm^−1^, 95% PI = −28.7 to 1.1; see details Supplementary material 3). The body condition of individuals at capture did not affect the birds’ probability of foraging outside the fjord on the first trip following their release (Table 2). There was no evidence for different patterns between years (interaction not significant, results not shown).

**Table 2.** Binomial generalized linear mixed model testing the effect of body condition at capture on the probability of foraging outside the fjord (during the first trip after the bird’s release). The colony (Ossian Sarsfjellet as reference level) and the year were added as fixed effects. Point estimates (mean; β) with 95% posterior uncertainty interval (*Post. Int.*) of the posterior probability distribution for each model parameter are indicated. The standard deviation of the random effect is 1.96. Significance (shown in bold) based on the nominal threshold of 0.05.

Predictors	β	*Post. Int. (95%)*
BC	-0.01	−0.04: 0.03
2016	−**4.42**	−**8.24:** −**2.20**
2017	−**5.02**	−**9.00:** −**2.27**
2018	−1.75	−4.09: 0.02
Colony (Ossian)	**2.01**	**0.15: 4.35**

**Fig. 4.**
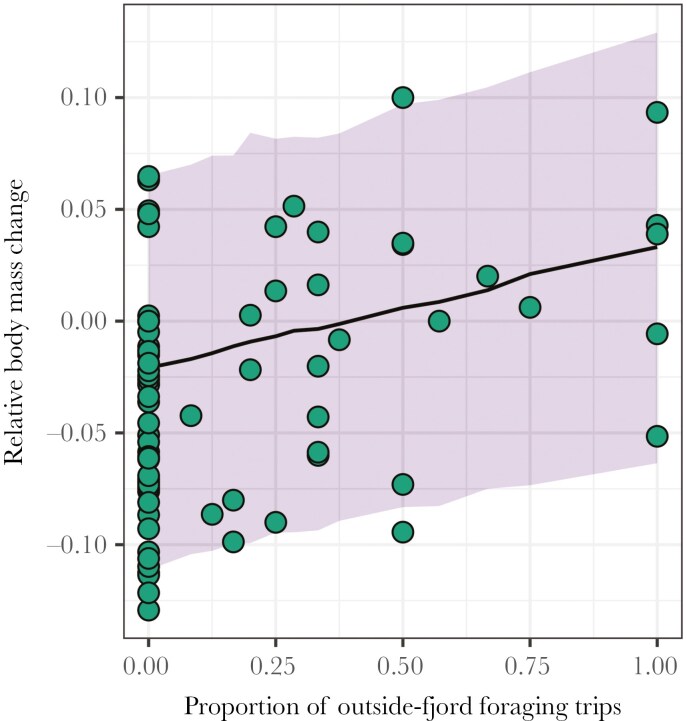
Interannual difference in the relative body mass change as a function of the proportion of outside-fjord foraging trips performed by chick-rearing kittiwakes breeding in Kongsfjorden, Svalbard. The regression line (median value of posterior samples) and its associated credible interval (90% Bayesian posterior uncertainty interval) were estimated from the corresponding Bayesian linear mixed model, with individual identity fitted as a random effect.

Birds tracked in 2017 experienced greater body mass loss (mean = −7.9%, 95% PI = −10.2 to −5.7) during the tracking period than birds tracked in 2016 (mean = −2.1%, 95% PI = −3.9 to −0.3) and 2018 (mean = −3.4, 95% PI = −5.9 to −0.7). Relative body mass changes were similar in 2016 and 2018 (mean difference = 1.3, 95% PI = −1.3 to 3.9). Individuals’ body mass change was positively related to the proportion of outside-fjord foraging trips during a whole tracking period (ie between capture and recapture, slope of 0.054; Table 3; Fig. 4). This suggests that chick-rearing adults performing more outside-fjord trips were more likely to improve their body condition than those foraging mainly within the fjord. This result was mostly driven by the season 2016 (ie excluding this season led to a non-significant effect of the proportion of outside-fjord foraging trips; results not shown). The effect size was nonetheless relatively small; foraging exclusively outside the fjord (ie a proportion of outside-fjord trips equal to 1) leads to an average body mass increase of 3.4% while foraging exclusively inside the fjord leads to an average body mass decrease of 2.0% (Fig. 4).

**Table 3.** Linear mixed model testing the effect of outside-fjord foraging trip (Proportion of trip out) on relative body mass change. The colony (Ossian Sarsfjellet as reference level) and the year were added as fixed effects. Point estimates (mean; β) with 95% posterior uncertainty interval (*Post. Int.*) of the posterior probability distribution for each model parameter are indicated. The standard deviation of the random effect and the standard deviation of the residuals equals, respectively, 0.01 and 0.04. Significance (shown in bold) based on the nominal threshold of 0.05.

Predictors	β	*Post. Int. (95%)*
**Prop**	**0.05**	**0.01: 0.10**
2016	−0.02	−0.04: 0.00
**2017**	−**0.08**	−**0.10:** −**0.06**
**2018**	−**0.03**	−**0.06:** −**0.01**
Colony (Ossian)	0.00	−0.02: 0.02

## Discussion

The investigation of foraging decisions is central to understanding how individuals respond to spatiotemporal variation in habitat quality and maximize fitness benefits (Orians and Pearson 1979; Schoener 1979). Adopting a bimodal foraging strategy, whereby birds alternate between long and short trips, may allow adults to balance their investments between survival (self-maintenance) and breeding performance (chick growth and survival). Adult kittiwakes breeding in Kongsfjorden adopted such a bimodal foraging strategy during the chick-rearing period in one of the three years under study, 2018, when zooplankton biomass was low inside the fjord. Contrary to our first prediction, we found no evidence that adult body condition affected the probability of foraging in sites located further away from the colony. However, adults could slightly improved their body condition when foraging outside the fjord compared to foraging exclusively inside. Overall, our results support the hypothesis that long-distance foraging trips could be, at least partially, used for self-maintenance but also suggest that the benefits of performing such trips may vary between years in central-place foragers like kittiwakes.

As suggested in other colonial seabirds such as Procellariiformes and Charadriiformes (eg Granadeiro et al. 1998; Steen et al. 2007), breeding kittiwakes may be more likely to perform long-distance foraging trips, and adopt a bimodal strategy, when the food supply close to their colony is poor. In our study system, kittiwakes can heavily forage at tidewater glacier fronts located within the fjord and a few kilometers away from their colony (Urbański et al. 2017; Bertrand et al. 2021a). However, annual variation in environmental conditions, including the level of zooplankton biomass in the fjord, appears to modulate the relative profitability of foraging habitats located within the fjord, including tidewater glacier fronts (Stempniewicz et al. 2017, 2021; Hamilton et al. 2019; Bertrand et al. 2021a). When the Atlantic inflow and zooplankton biomass levels in the fjord are relatively low, such as in 2018 in our study area, food available close to the breeding colony might be insufficient to fulfill all energetic requirements of both chick and adult kittiwakes. The bimodal foraging pattern observed in such year likely resulted from the spatial heterogeneity of the resource distribution, with optimal patches for foraging and offspring provisioning differing in localization (eg Steen et al. 2007; Ydenberg and Davies 2010; Welcker et al. 2012). Some variability in kittiwake foraging decisions remains unexplained—for example, why certain individuals undertook long foraging trips in 2016 and 2017, despite relatively high food abundance within the fjord during those years? The use of an annual proxy for food resource availability limits our capacity to assess the potential effects of intra-annual variations on individual foraging behavior. Additional data at a finer temporal scale would likely improve our ability to explain individual foraging decisions.

Bimodal foraging strategy has been reported previously in kittiwakes (Paredes et al. 2012; Christensen-Dalsgaard et al. 2018). For instance, kittiwakes breeding in Alaska performed long and short-distance trips along with a diurnal pattern in response to changing prey availability in the seascape (Kotzerka et al. 2010; *see also*Paredes et al. 2012). We did not detect such circadian pattern in departure time from the colony by the foraging kittiwakes in our study system (see Supplementary Information 4), suggesting that prey availability was not diurnally restricted within their feeding range. Based on four years of tracking, Christensen-Dalsgaard et al. (2018) observed that kittiwakes in mainland Norway were interspacing short- with long-distance foraging trips to feed at the Norwegian shelf break, a distant but predictable foraging habitat. They also found that birds with lower body condition tended to travel longer distances, suggesting that the use of distant patches likely promoted adult self-maintenance over chick provisioning (*see also*Ponchon et al. 2014). Although our results illustrate the energetic benefits of long-distance foraging trips for chick-rearing kittiwakes (ie an increase in adult body condition), we did not find that kittiwakes’ body condition modulated their decision to forage outside the fjord. A potential explanation could come from inter-individual differences in foraging behavior. In seabirds, it is common that individuals from a given population are characterized by different habitat or diet specialization (Ceia and Ramos 2015). Such variations in foraging behavior, and in how individuals respond to changes in the environment, are often linked to individual state variables such as age, experience or personality (Phillips et al. 2017; Harris et al. 2020). Such potential inter-individual differences in foraging behavior would obviously affect our ability to detect the effect of body condition on foraging trip distance. It has been shown in the Kongsfjorden kittiwake population that individuals respond differently to stress (ie lack of food; Schultner et al. 2013). All individuals are thus not expected to respond similarly to changes in their own body condition and some may endure variable thresholds in body condition to support their current reproductive performance. Such variation may thus obscure any relationship between an individual condition and the decision to improve its nutrient stores by foraging outside the fjord.

Moreover, the foraging decisions of a given individual may not only be dependent on its own body condition but also on the one of its offspring. In Manx shearwaters (*Puffinus puffinus*) for example, there is a negative relationship between chick condition and the duration of the parent’s foraging trips (Mas and Kölliker 2011). Such complex relationships between an individual’s foraging behavior, its state, and the state of its offspring may hinder the detection of any potential link between body condition at trip departure and foraging trip characteristics. Further studies that account for kittiwake chick condition would be needed to clarify these relationships.

The marine ecosystem of Kongsfjorden is changing rapidly towards a warmer and ice-free state, which is affecting the seabird community via the distribution and abundance of their prey (Descamps et al. 2017; Vihtakari et al. 2018; Hop et al. 2019; Descamps and Strøm 2021). Productivity in western Svalbard might increase if the Atlantification (ie increase influence of warm and saline Atlantic waters in the Arctic) trend persists (Csapó et al. 2021). Although glacier fronts are known to increase food availability for marine predators foraging in glacial fjords (Lydersen et al. 2014; Urbański et al. 2017), their profitability as foraging habitats might change rapidly once they recede above sea level (Hopwood et al. 2018; Halbach et al. 2019). Such changes could negatively affect prey availability near some seabird colonies and may potentially result in the need for more foraging trips outside the fjord to compensate for the loss of nearby foraging patches (Grémillet et al. 2015). Although kittiwakes can travel extensively while rearing their chicks (Christensen-Dalsgaard et al. 2018; Bertrand et al. 2021b), the disappearance of foraging areas close to the colony due to fast-changing glacial environments might still have important fitness consequences for breeding individuals and ultimately affect population trends (Descamps and Ramírez 2021).

## Supplementary Material

araf018_suppl_Supplementary_Materials

## Data Availability

Analyses reported in this article can be reproduced using the data provided by Bertrand et al. (2025).

## References

[CIT0001] Ameijeiras-Alonso J , Crujeiras RM, Rodríguez-Casal A. 2021. Multimode: an r package for mode assessment. J Stat Softw. 97:1–32. https://doi.org/10.18637/jss.v097.i09

[CIT0002] Baduini CL , Hyrenbach KD. 2003. Biogeography of procellariiform foraging strategies: does ocean productivity influence provisioning? Mar Ornithol. 31:101–112. https://doi.org/10.5038/2074-1235.31.2.570

[CIT0003] Ballard G , Dugger KM, Nur N, Ainley DG. 2010. Foraging strategies of Adélie penguins: adjusting body condition to cope with environmental variability. Mar Ecol Prog Ser. 405:287–302. https://doi.org/10.3354/meps08514

[CIT0006] Bertrand P , et al 2021a. Feeding at the front line: interannual variation in the use of glacier fronts by foraging black-legged kittiwakes. Mar Ecol Prog Ser. 677:197–208. https://doi.org/10.3354/meps13869

[CIT0004] Bertrand P , et al 2021b. Fine-scale spatial segregation in a pelagic seabird driven by differential use of tidewater glacier fronts. Sci Rep. 11:22109. https://doi.org/10.1038/s41598-021-01404-1 34764330 PMC8586018

[CIT0005] Bertrand P , et al 2025. Data from: Interannual variation in foraging decisions in chick-rearing black-legged kittiwakes. Behav Ecol. https://doi.org/10.5061/dryad.0cfxpnwcj PMC12322489 40766796

[CIT0007] Burr ZM , et al 2016. Later at higher latitudes: large-scale variability in seabird breeding timing and synchronicity. Ecosphere. 7:e01283. https://doi.org/10.1111/ecs2.2016.7.issue-5

[CIT0008] Canty A , Ripley B. 2024. boot: Bootstrap R (S-Plus) Functions. R package version 1.3-30.

[CIT0009] Ceia FR , Ramos JA. 2015. Individual specialization in the foraging and feeding strategies of seabirds: a review. Mar Biol. 162:1923–1938. https://doi.org/10.1007/s00227-015-2735-4

[CIT0010] Chaurand T , Weimerskirch H. 1994. The regular alternation of short and long foraging trips in the blue petrel *Halobaena caerulea*: a previously undescribed strategy of food provisioning in a pelagic seabird. J Anim Ecol. 63:275. https://doi.org/10.2307/5546

[CIT0011] Christensen-Dalsgaard S , May R, Lorentsen SH. 2018. Taking a trip to the shelf: behavioral decisions are mediated by the proximity to foraging habitats in the black-legged kittiwake. Ecol Evol. 8:866–878. https://doi.org/10.1002/ece3.3700 29375761 PMC5773323

[CIT0012] Clutton-Brock TH. 1988. Reproductive success: studies of individual variation in contrasting breeding systems. University of Chicago Press.

[CIT0013] Cottier F , et al 2005. Water mass modification in an Arctic fjord through cross-shelf exchange: the seasonal hydrography of Kongsfjorden, Svalbard. J Geophys Res Ocean. 110:C12005. https://doi.org/10.1029/2004JC002757

[CIT0014] Coulson JC. 2009. Sexing black-legged kittiwakes by measurement. Ringing Migr. 24:233–239. https://doi.org/10.1080/03078698.2009.9674397

[CIT0015] Coulson JC. 2011. The kittiwake. T. & A.D Poyser.

[CIT0016] Coulson JC , Macdonald A. 1962. Recent changes in the habits of the Kittiwake. Br Birds. 55:171–177. https://britishbirds.co.uk/journal/article/recent-changes-habits-kittiwake#:~:text=Recently%20formed%20colonies%20are%20noticeably,closer%20contact%20with%20human%20habitation

[CIT0017] Csapó HK , Grabowski M, Węsławski JM. 2021. Coming home - Boreal ecosystem claims Atlantic sector of the Arctic. Sci Total Environ. 771:144817. https://doi.org/10.1016/j.scitotenv.2020.144817 33736126

[CIT0018] Descamps S , et al 2017. Climate change impacts on wildlife in a High Arctic archipelago - Svalbard, Norway. Glob Chang Biol 23:490–502. https://doi.org/10.1111/gcb.13381 27250039

[CIT0019] Descamps S , Ramírez F. 2021. Species and spatial variation in the effects of sea ice on Arctic seabird populations. Divers Distrib. 27:2204–2217. https://doi.org/10.1111/ddi.13389

[CIT0020] Descamps S , Strøm H. 2021. As the Arctic becomes boreal: ongoing shifts in a high-Arctic seabird community. Ecology. 102:e03485. https://doi.org/10.1002/ecy.3485 34289096

[CIT0021] Dragańska-Deja K , Błaszczyk M, Deja K, Wesławski JM, Rodak J. 2020. Tidewater glaciers as feeding spots for the Black-legged kittiwake (*Rissa tridactyla*): a citizen science approach. Polish Polar Res. 41:69–93. https://doi.org/10.24425/ppr.2020.132570

[CIT0022] Elliott KH , Gaston AJ. 2015. Diel vertical migration of prey and light availability constrain foraging in an Arctic seabird. Mar Biol. 162:1739–1748. https://doi.org/10.1007/s00227-015-2701-1

[CIT0023] Fayet AL , Clucas GV, Anker-Nilssen T, Syposz M, Hansen ES. 2021. Local prey shortages drive foraging costs and breeding success in a declining seabird, the Atlantic puffin. J Anim Ecol. 90:1152–1164. https://doi.org/10.1111/1365-2656.13442 33748966

[CIT0024] Fryxell JM , Doucet CM. 1991. Provisioning time and central-place foraging in beavers. Can J Zool. 69:1308–1313. https://doi.org/10.1139/z91-184

[CIT0025] Gee S , Warzybok P, Johns ME, Jahncke J, Shaffer SA. 2024. Intra- and interannual variation in the foraging behavior of common Murres (*Uria aalge*) in the Central California current. J Exp Mar Bio Ecol. 575:152011. https://doi.org/10.1016/j.jembe.2024.152011

[CIT0026] Goodrich B , Gabry J, Ali I, Brilleman S. 2020. rstanarm: Bayesian applied regression modeling via Stan. R package version 2.21.1 https://mc-stan.org/rstanarm

[CIT0027] Goutte A , et al 2014. Annual variation in the timing of breeding, pre-breeding foraging areas and corticosterone levels in an Arctic population of black-legged kittiwakes. Mar Ecol Prog Ser. 496:233–247. https://doi.org/10.3354/meps10650

[CIT0028] Granadeiro JP , Nunes M, Silva MC, Furness RW. 1998. Flexible foraging strategy of Cory’s shearwater, *Calonectris diomedea*, during the chick-rearing period. Anim Behav. 56:1169–1176. https://doi.org/10.1006/anbe.1998.0827 9819333

[CIT0029] Grémillet D , et al 2015. Arctic warming: nonlinear impacts of sea-ice and glacier melt on seabird foraging. Glob Chang Biol. 21:1116–1123. https://doi.org/10.1111/gcb.12811 25639886

[CIT0030] Halbach L , et al 2019. Tidewater glaciers and bedrock characteristics control the phytoplankton growth environment in a fjord in the Arctic. Front Mar Sci. 6:254. https://doi.org/10.3389/fmars.2019.00254

[CIT0031] Hall P , York M. 2001. On the calibration of silverman’s test for multimodality. Stat Sin. 11:515–536. https://www.jstor.org/stable/24306875

[CIT0032] Hamilton CD , et al 2019. Contrasting changes in space use induced by climate change in two Arctic marine mammal species. Biol Lett. 15:20180834. https://doi.org/10.1098/rsbl.2018.0834 30836888 PMC6451376

[CIT0033] Harrell FEJ , Dupont C, Others. 2020. Hmisc: Harrell Miscellaneous. R package version 4.4-1. https://CRAN.R-project.org/package=Hmisc

[CIT0034] Harris SM , et al 2020. Personality predicts foraging site fidelity and trip repeatability in a marine predator. J Anim Ecol. 89:68–79. https://doi.org/10.1111/1365-2656.13106 31541578 PMC7004082

[CIT0035] Hegseth EN , et al 2019. Phytoplankton seasonal dynamics in Kongsfjorden, Svalbard and the adjacent shelf. In: Hop H, Wiencke C, editors. The ecosystem of Kongsfjorden, Svalbard. Advances in polar ecology 2. Springer. p. 173–227.

[CIT0036] Hop H , et al 2002. The marine ecosystem of Kongsfjorden, Svalbard. Polar Res. 21:167–208. https://doi.org/10.1111/j.1751-8369.2002.tb00073.x

[CIT0037] Hop H , et al 2019. Zooplankton in Kongsfjorden (1996–2016) in relation to climate change. In: Hop H., Wiencke C, editors. The ecosystem of Kongsfjorden, Svalbard. Advances in polar ecology 2. Springer. p. 229–300.

[CIT0038] Hopwood MJ , et al 2018. Non-linear response of summertime marine productivity to increased meltwater discharge around Greenland. Nat Commun. 9:3256. https://doi.org/10.1038/s41467-018-05488-8 30108210 PMC6092443

[CIT0039] Jacobs SR , et al 2012. Determining seabird body condition using nonlethal measures. Physiol Biochem Zool. 85:85–95. https://doi.org/10.1086/663832 22237292

[CIT0040] Jakob EM , Marshall SD, Uetz GW. 1996. Estimating fitness: a comparison of body condition indices. Oikos. 77:61. https://doi.org/10.2307/3545585

[CIT0041] Jakubas D , et al 2020. Flexibility of little auks foraging in various oceanographic features in a changing Arctic. Sci Rep. 10:8283. https://doi.org/10.1038/s41598-020-65210-x 32427941 PMC7237489

[CIT0042] Kacelnik A. 1984. Central Place Foraging in Starlings (*Sturnus vulgaris*). I. Patch Residence Time. J Anim Ecol. 53:283–299. https://doi.org/10.2307/4357

[CIT0043] Kacelnik A , Houston AI, Schmid-Hempel P. 1986. Central-place foraging in honey bees: the effect of travel time and nectar flow on crop filling. Behav Ecol Sociobiol. 19:19–24. https://doi.org/10.1007/bf00303838

[CIT0044] Kotzerka J , Garthe S, Hatch SA. 2010. GPS tracking devices reveal foraging strategies of Black-legged Kittiwakes. J Ornithol. 151:459–467. https://doi.org/10.1007/s10336-009-0479-y

[CIT0045] Lydersen C , et al 2014. The importance of tidewater glaciers for marine mammals and seabirds in Svalbard, Norway. J Mar Syst. 129:452–471. https://doi.org/10.1016/j.jmarsys.2013.09.006

[CIT0046] Markman S , Pinshow B, Wright J, Kotler BP. 2004. Food patch use by parent birds: to gather food for themselves or for their chicks? J Anim Ecol. 73:747–755. https://doi.org/10.1111/j.0021-8790.2004.00847.x

[CIT0047] Mas F , Kölliker M. 2011. Differential effects of offspring condition-dependent signals on maternal care regulation in the European earwig. Behav Ecol Sociobiol. 65:341–349. https://doi.org/10.1007/s00265-010-1051-8

[CIT0048] Murdoch DJ , Tsai YL, Adcock J. 2008. P-values are random variables. Am Stat. 62:242–245. https://doi.org/10.1198/000313008x332421

[CIT0049] Murphy ME. 1996. Nutrition and metabolism. In: C. Carey, editor. Avian energetics and nutritional ecology. Chapman and Hall. p. 31–60.

[CIT0050] Muth C , Oravecz Z, Gabry J. 2018. User-friendly Bayesian regression modeling: a tutorial with rstanarm and shinystan. Quant Meth Psych or TQMP. 14:99–119. https://doi.org/10.20982/tqmp.14.2.p099

[CIT0051] Newton I. 1989. Lifetime reproduction in birds. Academic Press.

[CIT0052] Nishizawa B , et al 2020. Contrasting assemblages of seabirds in the subglacial meltwater plume and oceanic water of Bowdoin Fjord, northwestern Greenland. ICES J Mar Sci. 77:711–720. https://doi.org/10.1093/icesjms/fsz213

[CIT0053] Orians GH , Pearson NE. 1979. On the theory of central place foraging. In: Horn DJ, Mitchell RD, Stairs GR. editors. Analysis of ecological systems. The Ohio State University Press. p. 154–177.

[CIT0054] Paredes R , et al 2012. Proximity to multiple foraging habitats enhances seabirds’ resilience to local food shortages. Mar Ecol Prog Ser. 471:253–269. https://doi.org/10.3354/meps10034

[CIT0055] Paredes R , et al 2014. Foraging responses of black-legged kittiwakes to prolonged food-shortages around colonies on the Bering Sea shelf. PLoS One. 9:e92520. https://doi.org/10.1371/journal.pone.0092520 24671108 PMC3966792

[CIT0056] Phillips RA , Lewis S, González-Solís J, Daunt F. 2017. Causes and consequences of individual variability and specialization in foraging and migration strategies of seabirds. Mar Ecol Prog Ser. 578:117–150. https://doi.org/10.3354/meps12217

[CIT0057] Ponchon A , et al 2014. When things go wrong: intra-season dynamics of breeding failure in a seabird. Ecosphere. 5:1–19. https://doi.org/10.1890/es13-00233.1

[CIT0058] Schoener TW. 1979. Generality of the size-distance relation in models of optimal feeding. Am Nat. 114:902–914. https://doi.org/10.1086/283537

[CIT0059] Schultner J , Kitaysky AS, Gabrielsen GW, Hatch SA, Bech C. 2013. Differential reproductive responses to stress reveal the role of life-history strategies within a species. Proc R Soc B Biol Sci. 280:20132090–20132011. https://doi.org/10.1098/rspb.2013.2090 PMC3790493 24089339

[CIT0060] Steen H , Vogedes D, Broms F, Falk-Petersen S, Berge J. 2007. Little auks (*Alle alle*) breeding in a High Arctic fjord system: bimodal foraging strategies as a response to poor food quality? Polar Res. 26:118–125. https://doi.org/10.1111/j.1751-8369.2007.00022.x

[CIT0061] Stempniewicz L , et al 2017. Marine birds and mammals foraging in the rapidly deglaciating Arctic fjord - numbers, distribution and habitat preferences. Clim Change. 140:533–548. https://doi.org/10.1007/s10584-016-1853-4

[CIT0062] Stempniewicz L , et al 2021. Advection of Atlantic water masses influences seabird community foraging in a high-Arctic fjord. Prog Oceanogr. 193:102549. https://doi.org/10.1016/j.pocean.2021.102549

[CIT0063] Svendsen H , et al 2002. The physical environment of Kongsfjorden–Krossfjorden, an Arctic fjord system in Svalbard. Polar Res. 21:133–166. https://doi.org/10.3402/polar.v21i1.6479

[CIT0064] Tverberg V , et al 2019. The Kongsfjorden transect: seasonal and inter-annual variability in hydrography. In: Hop H, Wiencke C, editors. The ecosystem of Kongsfjorden, Svalbard. Advances in polar ecology 2. Springer. p. 49–104.

[CIT0065] Urbański JA , et al 2017. Subglacial discharges create fluctuating foraging hotspots for seabirds in tidewater glacier bays. Sci Rep. 7:43999. https://doi.org/10.1038/srep43999 28266602 PMC5339806

[CIT0066] Varpe O , Tveraa T, Folstad I. 2004. State-dependent parental care in the Antarctic petrel: responses to manipulated chick age during early chick rearing. Oikos. 106:479–488. https://doi.org/10.1111/j.0030-1299.2004.13212.x

[CIT0067] Vihtakari M , et al 2018. Black-legged kittiwakes as messengers of Atlantification in the Arctic. Sci Rep. 8:1178. https://doi.org/10.1038/s41598-017-19118-8 29352216 PMC5775339

[CIT0068] Weimerskirch H , et al 1994. Alternate long and short foraging trips in pelagic seabird parents. Anim Behav. 47:472–476. https://doi.org/10.1006/anbe.1994.1065

[CIT0069] Weimerskirch H , Zimmermann L, Prince PA. 2001. Influence of environmental variability on breeding effort in a long-lived seabird, the yellow-nosed albatross. Behav Ecol. 12:22–30. https://doi.org/10.1093/oxfordjournals.beheco.a000374

[CIT0070] Welcker J , Beiersdorf A, Varpe O, Steen H. 2012. Mass fluctuations suggest different functions of bimodal foraging trips in a central-place forager. Behav Ecol. 23:1372–1378. https://doi.org/10.1093/beheco/ars131

[CIT0071] Welcker J , Steen H, Harding AMA, Gabrielsen GW. 2009. Sex-specific provisioning behaviour in a monomorphic seabird with a bimodal foraging strategy. Ibis (Lond 1859). 151:502–513. https://doi.org/10.1111/j.1474-919X.2009.00931.x

[CIT0072] Ydenberg RC. 1994. The behavioral ecology of provisioning in birds. Écoscience. 1:1–14. https://doi.org/10.1080/11956860.1994.11682222

[CIT0073] Ydenberg RC , Davies WE. 2010. Resource geometry and provisioning routines. Behav Ecol. 21:1170–1178. https://doi.org/10.1093/beheco/arq127

