# Supplementary material for: Interannual variation in foraging decisions in chick-rearing black-legged kittiwakes
Source: Behav Ecol. 2025 Mar 12;36(4):araf018. doi: 10.1093/beheco/araf018 (PMC12322489; doi:10.1093/beheco/araf018)
Supplement: araf018_suppl_Supplementary_Materials [file araf018_suppl_supplementary_materials.docx]

**Supplementary Information**

Interannual variation in foraging decisions in chick-rearing black-legged kittiwakes

[Supplementary Information 1 2](#_Toc180520085)

[Supplementary Information 2 4](#_Toc180520086)

[Supplementary Information 3 5](#_Toc180520087)

[Supplementary Information 4 6](#_Toc180520088)

[Cited Literature 7](#_Toc180520089)

# Supplementary Information 1

**Effect of GPS’s relative mass on birds’ behavior and weight change**

We distributed GPSs in random order in 2017 and 2018, letting us explore the effect of device weight on birds foraging behavior. In total, we collected 130 trips in 2017 and 75 in 2018.

We used a mixed regression model to evaluate whether GPS’s relative mass (% bird’s mass) was influencing the maximum range at which birds travelled for foraging. We also added the year as a predictor to control for the annual average use of extra-fjord foraging patches. Since environmental conditions differed among sampled years, we added the interaction term between the two predictors for testing whether the effect of GPS’s relative mass on birds’ foraging behavior varied between years. We moreover fitted individual identity as a random intercept to account for the non-independence of repeated individual measurements. Modelling was performed under the Bayesian framework using the RSTANARM package (Goodrich et al. 2020). Please refer to the Methods section of the manuscript for all details about the parameterization.

**Table S1.1.** Estimates for the two mixed regression models testing the effect of GPS’s relative mass and year on the birds’ maximum range travelled during their tracking period. Point estimates (mean and associated posterior standard deviation) with 95% posterior uncertainty interval (Post. Int.) of the posterior probability distribution for each model parameter are indicated. Both main effects and interaction models are presented.

|  | **Main effect model** | | **Interaction model** | |
| --- | --- | --- | --- | --- |
| *Coefficient* | *Estimates ± SD* | *Post. Int. (95%)* | *Estimates ± SD* | *Post. Int. (95%)* |
| Intercept | 2.34 *±* 0.75 | 0.86 to 3.83 | 1.62 *±* 1.40 | -1.13 to 4.39 |
| GPS | -0.09 *±* 0.20 | -0.48 to 0.30 | 0.11 *±* 0.38 | -0.65 to 0.86 |
| Year (2018) | 1.02 *±* 0.39 | 0.27 to 1.80 | 1.99 *±* 1.60 | -1.17 to 5.13 |
| GPS x Year (2018) |  |  | -0.26 *±* 0.42 | -1.09 to 0.57 |

We then used a linear mixed model to evaluate whether the GPS’s relative mass was modulating the relative body mass change of birds during their tracking period (see *Materials and Methods* for details on variables). As in the previous model, the year and interaction term were also included. We also fitted individual identity as a random intercept to account for the non-independence of repeated individual measurements.

**Table S1.2.** Estimates for the two linear mixed regression models testing the effect of GPS’s relative mass and year on the birds’ relative body mass change during their tracking period. Point estimates (mean and associated posterior standard deviation) with 95% posterior uncertainty interval (Post. Int.) of the posterior probability distribution for each model parameter are indicated. Both main effects and interaction models are presented.

|  | **Main effect model** | | **Interaction model** | |
| --- | --- | --- | --- | --- |
| *Coefficient* | *Estimates ± SD* | *Post. Int. (95%)* | *Estimates ± SD* | *Post. Int. (95%)* |
| Intercept | -0.09 ± 0.03 | -0.14 to -0.04 | -0.02 ± 0.05 | -0.12 to 0.08 |
| GPS | 0.00 ± 0.01 | -0.01 to 0.02 | -0.02 ± 0.01 | -0.04 to 0.01 |
| Year (2018) | 0.07 ± 0.02 | 0.04 to 0.10 | -0.03 ± 0.06 | -0.14 to 0.09 |
| GPS x Year (2018) |  |  | 0.03 ± 0.02 | 0.00 to 0.06 |

Overall, we detected no effect of the GPS’s relative mass on either birds’ maximum range or their relative body mass change during their tracking periods (*i.e.*, 95% posterior uncertainty interval of relevant estimates overlap zero; Table S1.1 & S1.2).

Supplementary Information 2
**Assessment of probability distributions by a resampling approach**

We assessed bimodality in maximum foraging ranges by using a resampling approach. This approach had the benefit of considering the probability of including all trips made by the 71 tracked individuals. For each year, we randomly selected one trip per individual and computed the Hartigans’ dip test statistic and its corresponding p-values. This process was iterated 999 times, resulting in a probability distribution for each year studied.

A probability distribution is assumed to be uniform under the null hypothesis (Murdoch et al. 2008). In the Hartigans’ dip test, the null hypothesis assumes an unimodal distribution (Ameijeiras-Alonso et al. 2021). A distribution of p-values skewed to the left would indicate support for this null hypothesis. In contrast, a distribution skewed to the right would indicate support for the alternative hypothesis, here, in the context of the Hartigans’ dip test, the presence of multimodality.

Based on the resampling procedure, the probability distribution of 2018 is highly right-skewed and clearly rejects the null hypothesis *i.e.*, that the maximum foraging range distribution is unimodal (Fig. S2.1). In 2017, the uniform distribution fits the expectations under the null hypothesis, which suggests that the maximum foraging range distribution of this year is unimodal. In 2016, the probability distribution is highly left-skewed, supporting the null hypothesis. This large bias towards 1 may also suggest that the maximum foraging range distribution is not uniform but without a distinct mode. This bias could also stem from a lack of sensitivity of the test or that some hypotheses (*i.e.*, other than modality) are not verified.

**
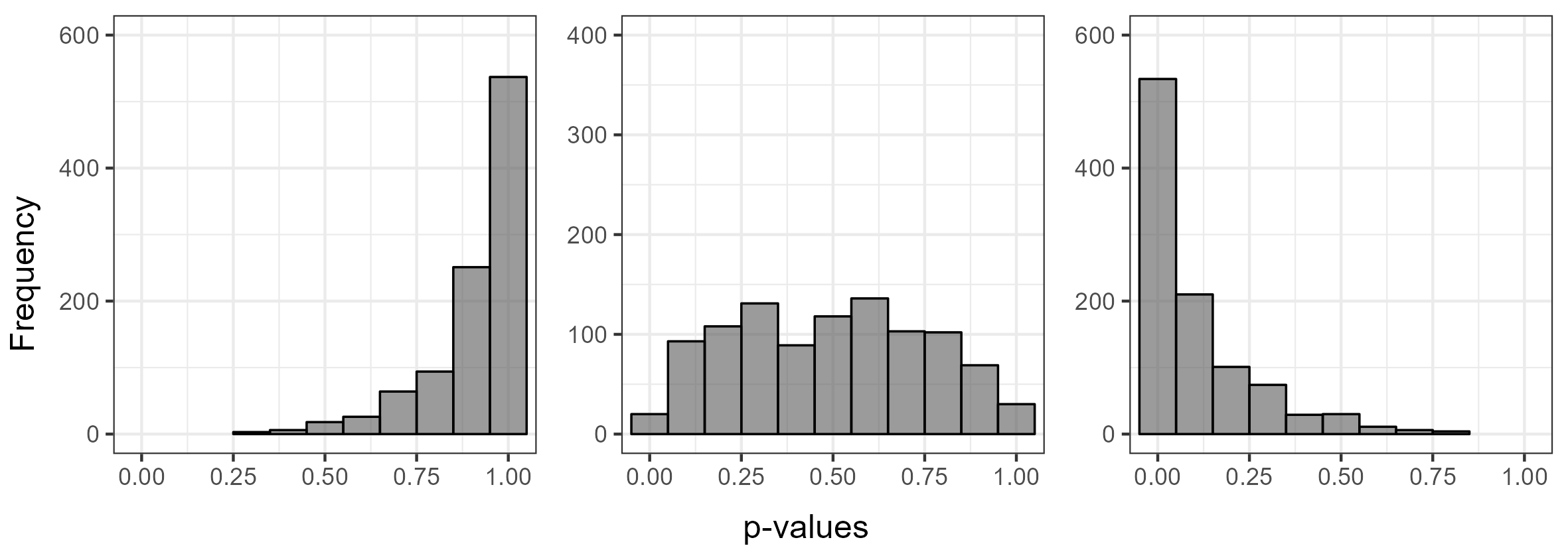
**

**Figure S2.1.** Annual probability distribution derived from Hartigan’s dip test using a random sampling approach. Distribution of 2016 (left), 2017 (center) and 2018 (right) are shown.

Supplementary Information 3
**Interannual variation in individual body condition**

We used a mixed modelling approach to investigate whether individual body conditions were consistent over the years (see Methods for details on how body conditions were calculated). In addition to the year, the colony was also added as a predictor. We fitted individual identity as a random intercept to account for the non-independence of repeated individual measurements. Again, modelling was performed under the Bayesian framework using the RSTANARM package (Goodrich et al. 2020). Please refer to the Methods section of the manuscript for all details about the parameterization.

In 2017, the body condition of individuals breeding in Kongsfjorden was higher than in both 2016 (mean = 31.2, 95%CI = 15.5 to 47.0) and 2018 (mean = 35.9, 95%CI = 18.7 to 53.5), while the body conditions observed in 2016 and 2018 were not significantly different (mean = 4.7, 95%CI = -11.1 to 20.5) (Figure S3.1).

**
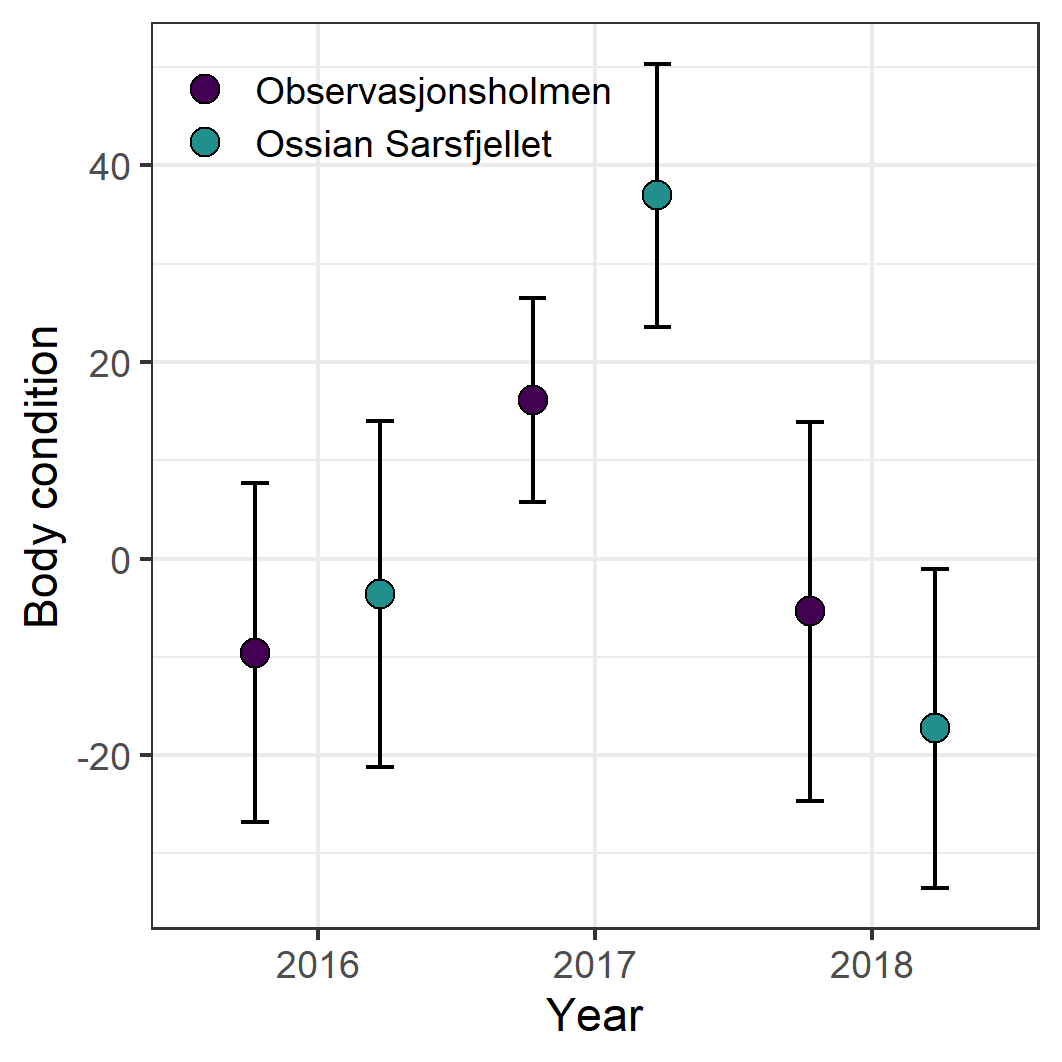
**

**Figure S3.1.** Interannual variation in individual body condition for each colony (Observasjonsholmen: purple, Ossian Sarsfjellet: green) between 2016 and 2018.

# Supplementary Information 4

**Temporal distribution of birds foraging trips**

We used a generalized linear mixed model with binomial distribution to test whether the time (*i.e.*, hour; distribution shown in Figure S4.1) of departure to foraging trip affected the probability of using extra-fjord foraging areas. Foraging areas were coded as binary, where 0 was assigned to trips where birds remained inside fjord boundaries to forage and 1 to trips where birds flew out of the fjord. We added year and colony as predictors, and the individual identity was fitted as a random intercept.


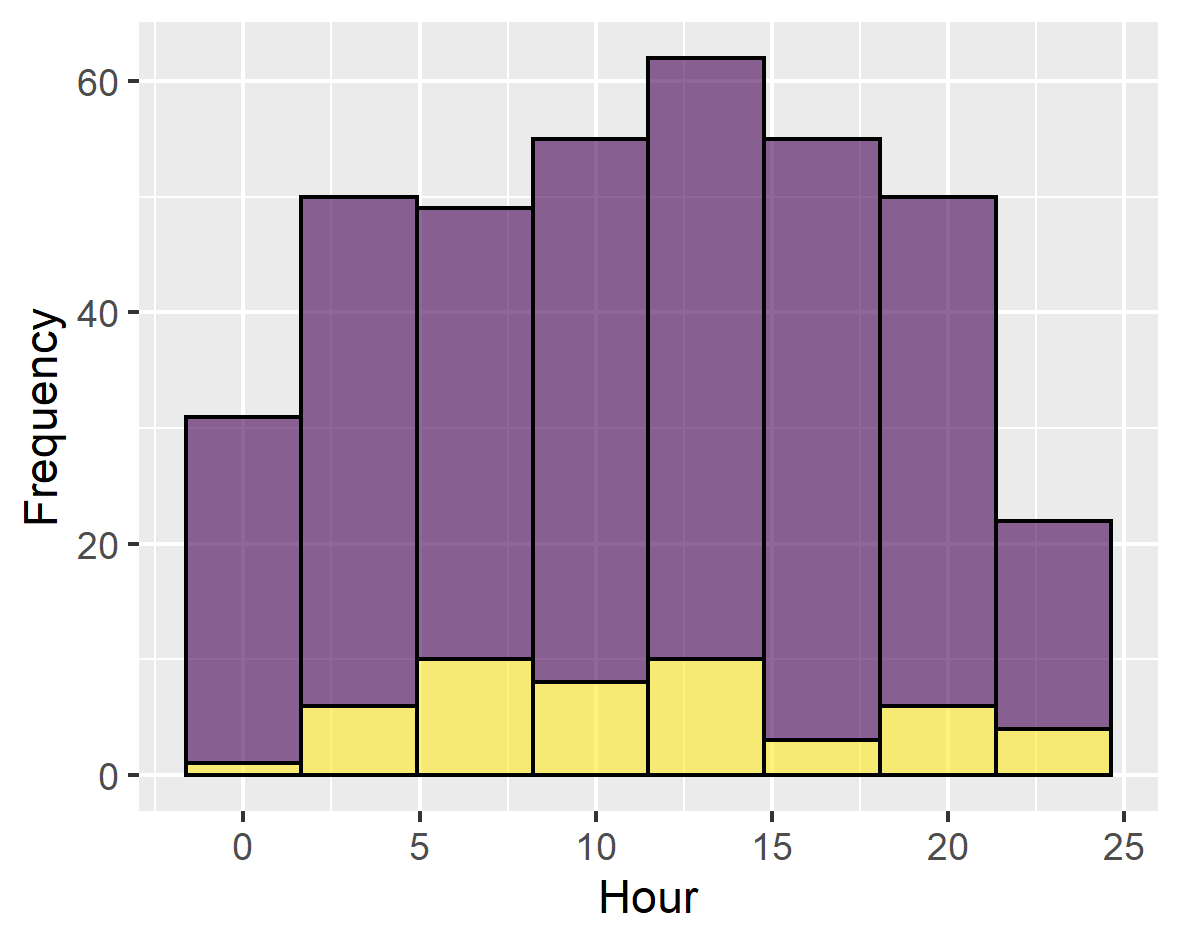


**Figure S4.1.** Frequencies of time of day (hour) when kittiwakes left the colony to forage (purple = intra-fjord; yellow = extra-fjord foraging trips).

**Table S4.1.** Binomial generalized mixed model testing the effect of the time (hour) of departure to foraging trip on the probability of performing extra-fjord foraging trips. The colony (using 2016 as the reference level) and the year were also included as fixed effects and the individual ID as random effects. Model estimates (log-odds) and associated 95% confidence intervals are indicated.

| Model | Log-Odds | Conf. Int (95%) |
| --- | --- | --- |
| Hour | 0.01 | -0.04 to 0.07 |
| Colony | 0.74 | -0.02 to 1.56 |
| Year 2017 | -1.34 | -2.65 to -0.26 |
| Year 2018 | 1.40 | 0.62 to 2.29 |

Time (hour) of departure to foraging trip did not modulate the probability of using extra-fjord foraging patches in kittiwakes breeding in Kongsfjorden (*i.e.*, 95% confidence interval of the predictor estimate overlapped zero; Table S4.1). This suggests that prey availability was not differing in time between patches inside and outside the fjord.

# Cited Literature

Ameijeiras-Alonso J, Crujeiras RM, Rodríguez-Casal A. 2021. Multimode: An r package for mode assessment. J Stat Softw. 97(9):1–32. doi:10.18637/jss.v097.i09.

Goodrich B, Gabry J, Ali I, Brilleman S. 2020. rstanarm: Bayesian applied regression modeling via Stan. R package version 2.21.1 https://mc-stan.org/rstanarm.

Murdoch DJ, Tsai YL, Adcock J. 2008. P-values are random variables. Am Stat. 62(3):242–245. doi:10.1198/000313008X332421.
